# Epidemiology of Ebola virus disease transmission among health care workers in Sierra Leone, May to December 2014: a retrospective descriptive study

**DOI:** 10.1186/s12879-015-1166-7

**Published:** 2015-10-13

**Authors:** Olushayo Olu, Brima Kargbo, Sarian Kamara, Alie H. Wurie, Jackson Amone, Louisa Ganda, Bernard Ntsama, Alain Poy, Fredson Kuti-George, Etsub Engedashet, Negusu Worku, Martin Cormican, Charles Okot, Zabulon Yoti, Kande-Bure Kamara, Kennedy Chitala, Alex Chimbaru, Francis Kasolo

**Affiliations:** World Health Organization (WHO) Intercountry Support Team for Eastern and Southern Africa, Harare, Zimbabwe; Ministry of Health and Sanitation, Freetown, Sierra Leone; Ministry of Health, Kampala, Uganda; WHO, Freetown, Sierra Leone; WHO Intercountry Support Team West Africa, Ouagadougou, Burkina Faso; WHO African Regional Office (AFRO), Brazzaville, Congo; School of Medicine, National University of Ireland Galway, Galway, Ireland; WHO, Kampala, Uganda; WHO, Geneva, Switzerland; WHO, Nairobi, Kenya

**Keywords:** Ebola, Virus, Transmission, Health care, Workers

## Abstract

**Background:**

Anecdotal evidence suggests that much of the continuing infection of health care workers (HCWs) with Ebola virus during the current outbreak in Sierra Leone has occurred in settings other than Ebola isolation units, and it is likely that some proportion of acquisition by HCWs occurs outside the workplace. There is a critical need to define more precisely the pathways of Ebola infection among HCWs, to optimise measures for reducing risk during current and future outbreaks.

**Methods:**

We conducted a retrospective descriptive study of Ebola acquisition among health workers in Sierra Leone during May–December 2014. The data used were obtained mainly from the national Ebola database, a cross-sectional survey conducted through administration of a structured questionnaire to infected HCWs, and key informant interviews of select health stakeholders.

**Results:**

A total of 293 HCWs comprising 277 (95 %) confirmed, 6 (2 %) probable, and 10 (3 %) suspected cases of infection with Ebola virus were enrolled in the study from nine districts of the country. Over half of infected HCWs (153) were nurses; others included laboratory staff (19, 6.5 %), doctors (9, 3.1 %), cleaners and porters (9, 3.1 %), Community Health Officers (8, 2.7 %), and pharmacists (2, 0.7 %). HCW infections were mainly reported from the Western Area (24.9 %), Kailahun (18.4 %), Kenema (17.7 %), and Bombali (13.3 %) districts. Almost half of the infected HCWs (120, 47.4 %) believed that their exposure occurred in a hospital setting. Others believed that they were exposed in the home (48, 19 %), at health centres (45, 17.8 %), or at other types of health facilities (13, 5.1 %). Only 27 (10.7 %) of all HCW infections were associated with Ebola virus disease (EVD) isolation units. Over half (60 %, 150) of infected HCWs said they had been trained in infection prevention and control prior to their infection, whereas 34 % (85) reported that they had not been so trained.

**Conclusions:**

This study demonstrated the perception that most HCW infections are associated with general health care and home settings and not with dedicated EVD settings, which should provide substantial reassurance to HCWs that measures in place at dedicated EVD facilities generally provide substantial protection when fully adhered to.

## Background

Ebola virus disease (EVD) is an infectious disease caused by a virus belonging to the Filoviridae family of RNA viruses. Ebola virus is transmitted from human to human, primarily through close contact with the body fluids or corpse of an EVD patient or contact with contaminated environments. Transmission appears most likely with advanced disease when viral load is high and the patient has uncontrolled diarrhoea, vomiting, and bleeding [1]. The ongoing EVD outbreak in West Africa that began in December 2013 is unprecedented. As of 4 February 2015, a total 22,495 cases (confirmed, probable, and suspected) and 8981 deaths (case fatality rate, (CFR) 40 %) had been reported in nine countries, namely, Guinea, Liberia, Mali, Nigeria, Senegal, Sierra Leone, Spain, the United Kingdom and United States [2]. The Government of Sierra Leone (GoSL), through its Ministry of Health and Sanitation (MOHS), declared an EVD outbreak in the country on 25 May 2014 following laboratory confirmation of a suspected case in the eastern district of Kailahun, which borders Guinea and Liberia. As of 4 February 2014, a total 10,740 cases (8059 confirmed, 287 probable, and 2394 suspected) and 3276 deaths (CFR 30.5 %) had been recorded in the country [2].

Health care delivery settings play major roles in the propagation of EVD outbreaks [3]. In such settings, the risk is high for health care workers (HCWs) to come in direct contact with patients with advanced EVD, their body fluids, and contaminated environments, if strict infection prevention and control (IPC) is not practiced [4]. Nursing a patient or assisting with childbirth are some of the major risk factors for acquisition of EVD in health facility settings [5, 6]. In Sierra Leone, the estimated cumulative incidence rate of EVD is 8285 per 100,000 population among HCWs, more than 100 times higher than that of the general population (incidence of 80.4 per 100,000) [7]. This high EVD incidence rate reiterates the vulnerability of HCWs to nosocomial transmission of the disease. HCW infections in this outbreak have exceeded those recorded in all other outbreaks in Africa combined [8]. In the first 9 months of the outbreak, 318 HCWs had been infected in Guinea, Liberia, and Sierra Leone, with 152 deaths (CFR 48 %) [2]. By February 2015, these figures had almost tripled, with an estimated 822 HCW infections and 488 deaths (CFR 59 %) in the three countries [2]. As of that date, more than 250 HCWs had been infected in Sierra Leone, with mortality estimated at 77 %. This represents serious deterioration of already precarious human resources for health (HRH) situation in the country [8, 9]. The resulting apprehension and loss of morale among HCWs has caused many to abandon their work, thereby amplifying the impact of EVD on health service delivery in Sierra Leone. General EVD prevention and control efforts were strengthened in the country during August 2014 and basic IPC training for HCWs was intensified beginning in September 2014.

Because EVD is associated with life-threatening disease and death, it represents a major risk for HCWs and a threat to the operation of health care systems. Anecdotal evidence suggests that much of the ongoing acquisition of infection during the current outbreak has occurred in settings other than EVD isolation units, and it is likely that some proportion of acquisition by HCWs occurs outside the workplace [7]. There is therefore a critical need to define more precisely the pathways of EVD acquisition by HCWs, to optimise measures for reducing risks during the current and future outbreaks.

In this study, we investigated the pattern of EVD infection among HCWs during the 2014 EVD outbreak in Sierra Leone. The objectives were to describe the epidemiology of EVD infection among HCWs and clarify the locations, sources, and modes of HCW exposure to and acquisition of EVD in the country. We also assessed self-reported IPC training, knowledge, and practice among infected HCWs.

## Methods

We conducted a retrospective descriptive study of EVD acquisition among HCWs in Sierra Leone from May to December 2014. We obtained the study data from three main sources: 1) the national EVD database, which is an Epi Info application called Viral Hemorrhagic Fever (VHF), developed by the Centers for Disease Control and Prevention (CDC) [10]; 2) a cross-sectional survey conducted through administration of a structured questionnaire to infected HCWs; 3) key informant interviews of select health stakeholders in the affected districts.

We extracted case investigation data for all HCWs listed in the national VHF database from May through December 2014. A HCW is defined as any person involved in the promotion, protection, or improvement of the health of the population [11]. Based on this definition, we included all categories of workers who are directly or indirectly involved in EVD health services delivery. These include doctors, clinical officers, nurses, community health officers, surveillance officers, ambulance drivers, and support staff (hygienists, porters, and others). All HCWs in the database were listed for study. We descriptively analysed the HCW data using Epi Info 7. Preliminary analysis revealed underestimation of HCW infections in several districts and missing data, such as location, mode, and type of exposure, which are required to better characterise EVD transmission among HCWs.

To collect additional data, a structured questionnaire was developed, pre-tested, and administered to all HCWs in the national database, as well as to other infected HCWs identified by the District Health Management Teams (DHMTs). The questionnaire contained 42 questions and sub-questions that were categorised into four main sections, i.e., HCW identity, exposure, and knowledge and practice of IPC. Six data collectors were identified (three from the WHO data management team, who are co-authors of this paper, and three external candidates) and trained on the study protocol and questionnaire administration, to ensure that the questionnaire was administered in a uniform manner. The data collectors then pre-tested the questionnaire and administered the final version to respondents.

Nine districts that reported HCW infections during the study period were covered. All surviving HCWs were interviewed in person. In cases of HCWs who had died, next of kin or close associates were interviewed. A relative or close associate was defined as a spouse, parent, brother or sister who was close to or lived with the deceased HCW. To ensure completeness of data, colleagues working in the same health facility as deceased HCWs were also interviewed, in order to obtain workplace-related information that could not be provided by relatives or associates.

Qualitative data were collected through key informant interviews of District Medical Officers (DMOs) and Matrons at district hospitals in selected districts. A purposive sampling method was used to identify four high transmission districts, namely, Kenema, Port Loko, Bombali and Tonkolili. A fifth district, Bonthe, which had not reported any HCW infections at the time of our study, was also selected to better understand the challenges of EVD prevention in non-transmission districts. A key informant interview guide was developed by the research team and used to interview DMOs and Matrons in these districts. The key informant interview guide had five main questions that explored the availability and implementation of IPC policies in the districts, the perceived causes of HCW infection, the challenges associated with ensuring HCW safety, and what could be done to prevent HCW infection in the future. The key informant interviews were conducted by two members of the research team; six key informant interviews were conducted in total.

Survey data were entered into a Microsoft Excel database, cleaned, and then exported into Epi Info 7. In the first stage of analysis, we conducted univariate analyses on all variables in the database. In the second stage, we performed detailed descriptive analyses on the key variables. The descriptive analyses included the distribution of cases and deaths for person, location, time, IPC knowledge, disease outcome, location and type of exposure. We obtained the total number of HCWs in Sierra Leone from the National Health Sector Strategic Plan 2010–2015 [12] and used this to calculate the rate of EVD infection among HCWs. The key informant interview data were transcribed, and responses were coded and collated [13]. Some respondents did not respond to all the questions, which explains the differences in denominators in some results.

This study was part of extended epidemiological investigations to provide scientific evidence for initiating tailored interventions to control the EVD outbreak in Sierra Leone. The study was approved by the MOHS. All data presented herein are anonymous. In collaboration with the MOHS and DHMTs, the data collectors identified the affected HCWs, explained the study purpose, and obtained their verbal consent to participate in the study.

## Results

**Fig. 1:**
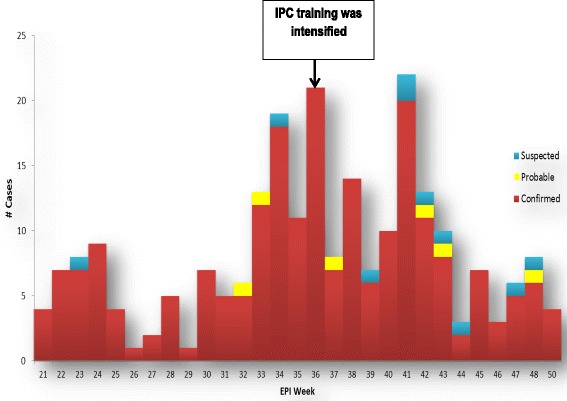
Epidemic curve of EVD among HCW in Sierra Leone - May to December 2014

A total of 293 HCWs comprising 277 (95 %) confirmed, six (2 %) probable, and 10 (3 %) suspected cases were enrolled in the study from nine districts of Sierra Leone: Bo, Bombali, Kailahun, Kenema, Kono, Moyamba, Port Loko, Tonkolili, and the Western Area (Table 1). Based on an estimated total of 2435 HCWs in the country, 12 % were infected during the study period. More males were infected than females (Table 2). The most affected HCW age groups were 26–35 and 36–45 years old (Table 2), with mean, median and range of the age groups at 40.3, 39 and 20–70 years, respectively. Over half the infected HCWs were nurses. Other groups included laboratory staff (19, 6.5 %), doctors (9, 3.1 %), cleaners and porters (9, 3.1 %), Community Health Officers (8, 2.7 %), and pharmacists (2, 0.7 %). HCW infections were mainly reported from the Western Area, Kailahun, Kenema and Bombali districts (Table 1). Port Loko, the district with the second highest number of reported EVD cases in the outbreak, accounted for only 6.8 % (20) of all HCW infections. The first HCW infections were reported in epidemiological week 21 of 2014 (week ending 24 May 2014). Subsequently, two peaks were observed from epidemiological weeks 33 through 38 (11 August through 21 September 2014)) and 41 through 43 (6 to 26 October 2014). A smaller peak was observed in epidemiological week 48 (24 through 30 November 2014) (Fig. 1).

**Table 1:** Distribution of infected HCWs in Sierra Leone, May through December 2014

District	Case classification(n)				Outcome of infection (%)	
	Suspected	Probable	Confirmed	Total	Survived	Died
Bo	4	0	14	18 (6.1 %)	50 %	50 %
Bombali	0	0	39	39 (13.3 %)	15 %	85 %
Kailahun	1	4	49	54 (18.4 %)	19 %	81 %
Kenema	0	0	52	52 (17.7 %)	19 %	81 %
Kono	0	0	10	10 (3.4 %)	29 %	71 %
Moyamba	3	0	3	6 (2 %)	50 %	50 %
Port Loko	0	0	20	20 (6.8 %)	25 %	75 %
Tonkolili	1	1	19	21 (7.2 %)	29 %	71 %
Western Area	1	1	71	73 (24.9 %)	23 %	77 %
Total	10	6	277	293	23 %	77 %

**Table 2:** Age and sex distribution of EVD-infected HCWs in Sierra Leone, May through December 2014

Age group (years)	Gender (%)		Total (%)
	Male	Female	
18 – 25	11 (79 %)	3 (21 %)	14 (5.0 %)
26 – 35	56 (59 %)	39 (41 %)	95 (33.7 %)
36 – 45	44 (46 %)	51 (54 %)	95 (33.7 %)
46 – 55	32 (62 %)	20 (38 %)	52 (18.4 %)
56 – 65	16 (64 %)	9 (36 %)	25 (8.9 %)
66 – 75	1 (100 %)	0 (0 %)	1 (0.4 %)
Total	160 (56.7 %)	122 (43.3 %)

**Fig. 2:**
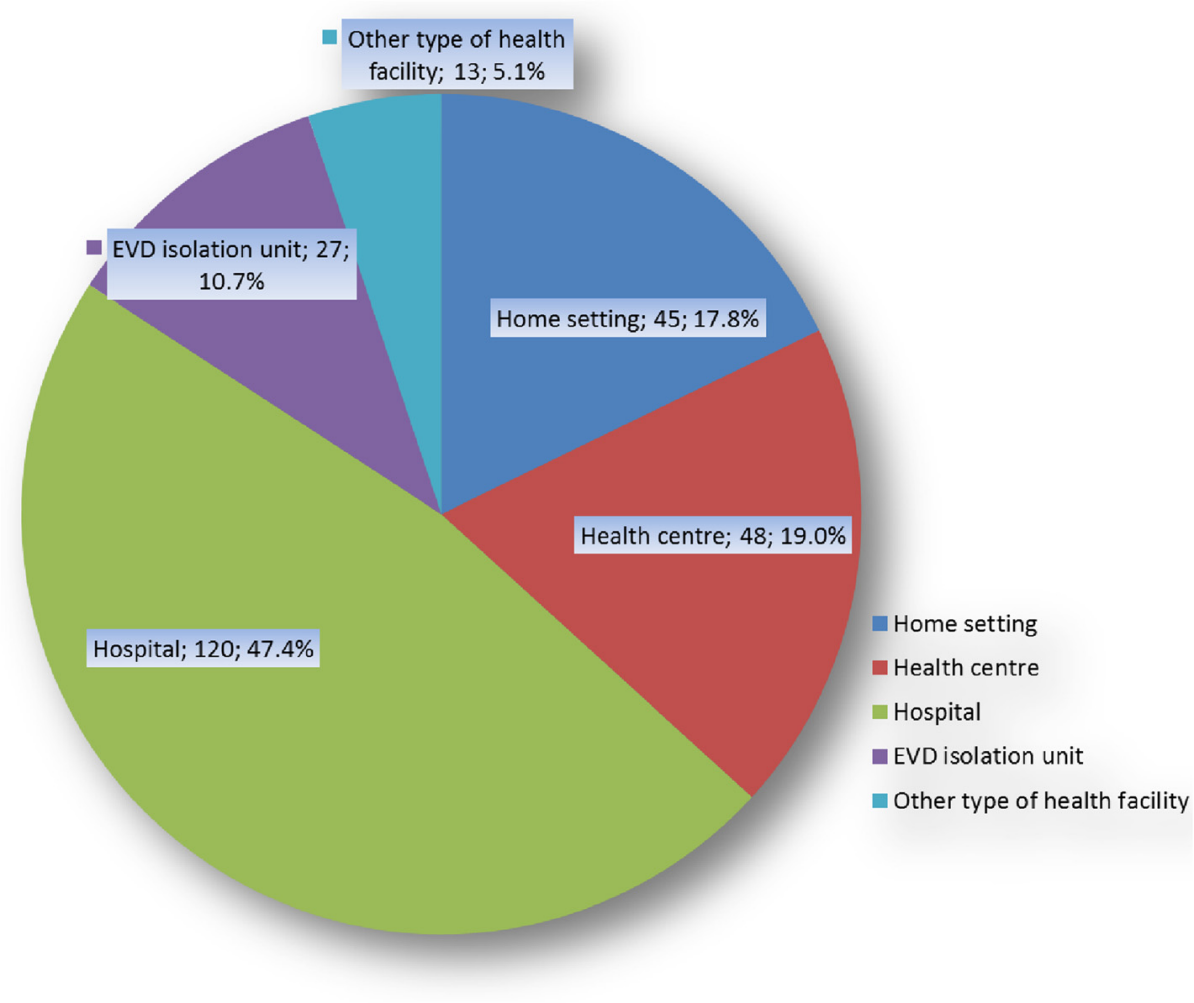
Reported association of infection with working in a particular health care setting

Almost half the infected HCWs believed that their exposure had occurred in a hospital setting. Others believed that they were exposed in the home (48, 19 %), at health centres (45, 17.8 %), or at other types of health facilities (13, 5.1 %). Only 10.7 % of all HCW infections were associated with EVD isolation units (Fig. 2). Among those that reported exposure to the virus in the home setting, the respondents said that the patient they had physical contact with was a family member (41 %), another HCW (20 %), or a friend (9 %). Nearly all (91 %) of infected HCWs recalled having been in contact with an EVD patient within the 3 weeks prior to onset of their symptoms. Of those who reported contact with an EVD patient prior to symptom onset, 35 % (88) and 19 % (47) said they had come in contact with confirmed and suspected cases, respectively, whereas 46 % (117) did not know the status of the patient with whom they had contact. In this context, “contact” was defined as having unprotected bodily contact with a suspected, probable, or confirmed EVD case during patient care, in either a health facility or home setting. A total 87 % of respondents said that they were aware of the specific exposure that had resulted in their infection, whereas 13 % were unable to pinpoint the specific exposure. More than half (57 %, 138) of the infected HCWs were aware of the exact time of their exposure/infection, whereas 106 (43 %) reported that they were unaware of this time.

Concerning mode of exposure, 55 % of respondents said that exposure was through general medical and nursing care of infected persons. Other modes of infection were direct body contact with an EVD patient, contact with a contaminated surface, transport of an EVD patient, or during removal of personal protective equipment (PPE) (Table 3). The most common types of exposure were parenteral (e.g., needle stick injury) and direct contact of mucous membranes with infectious material (Table 3). Blood and body fluid containing visible blood were the two most common types of infectious materials involved, and most respondents identified their hands as the body part that had been contaminated (Table 3). Although a high percentage of infected HCWs (57 %) presented to a treatment centre within 3 days of symptom onset, a sizable percentage (43 %) presented 4 or more days after manifesting symptoms (Table 4). Many respondents reported that they had performed hand hygiene at the time of exposure (Table 5).

**Table 3:** Mode and type of exposure of infected HCWs

Exposure variable	Response	Number of observation	Percentage
Mode of exposure (*n* = 246)	Provision of general medical and nursing care	136	55.3 %
	Body contact	71	28.9 %
	Contact with contaminated surface	17	6.9 %
	Transportation of patient to EVD isolation unit	10	4.1 %
	During removal of PPE	7	2.8 %
	Not wearing PPE	1	0.4 %
	Handling EVD sample (blood or corpse)	4	1.6 %
Type of exposure (*n* = 289)	Parenteral (needle stick injury,)	92	32 %
	Direct contact of infectious material with mucous membrane	33	11 %
	Direct contact of infectious material with non-intact skin such as cuts	21	7 %
	Splash	21	7 %
	Others	122	42 %
Type of infectious material (*n* = 208)	Blood	68	32.7 %
	Body fluid with visible blood	91	43.8 %
	Vomitus	30	14.4 %
	Faeces	3	1.4 %
	Urine	3	1.4 %
	Internal body fluid (cerebrospinal, amniotic, peritoneal, pericardial Etc.)	12	5.8 %
	Vaginal secretion	1	0.5 %
Contaminated body part (*n* = 292)	Face	21	7.2 %
	Hand	237	81.2 %
	Arm	13	4.5 %
	Leg	2	0.7 %
	Foot	4	1.4 %
	Torso	3	1.0 %
	Others	12	4.1 %

**Table 4:** Time interval between onset of symptoms and admission to a treatment centre

Time interval in days	No and percentage of observation		
	Alive (%)	Dead (%)	Total (% time interval)
0 – 3	33 (26 %)	92 (74 %)	125 (57 %)
4 – 7	16 (25 %)	47 (75 %)	63 (29 %)
8 – 11	7 (28 %)	18 (72 %)	25 (11 %)
12 and above	1 (17 %)	5 (83 %)	6 (3 %)
Total	57 (26 %)	162 (74 %)	219 (100 %)

**Table 5:** Training of infected HCWs on IPC and availability of IPC facilities and policies at time of HCW infection (by work location)

	Responses	Number of observations					
	Responses	Home setting	Health care centre	Hospital setting	Isolation unit	Other units	Total
Infected HCW trained on IPC (*n* = 249)	Yes	24 (53 %)	14 (30 %)	80 (68 %)	24 (89 %)	8 (62 %)	150 (60 %)
	No	19 (42 %)	27 (57 %)	33 (28 %)	3 (11 %)	3 (23 %)	85 (34 %)
	Don’t Know	2 (4 %)	6 (13 %)	4 (3 %)	0 (0 %)	2 (15 %)	14 (6 %)
	Total	45	47	117	27	13	249
Availability of IPC policy (*n* = 250)	Yes	6 (13 %)	27 (56 %)	47 (40 %)	20 (74 %)	5 (38 %)	105 (42 %)
	No	35 (78 %)	16 (33 %)	66 (56 %)	5 (19 %)	6 (46 %)	128 (51 %)
	Don’t Know	4 (9 %)	5 (10 %)	4 (3 %)	2 (7 %)	2 (15 %)	17 (7 %)
	Total	45	48	117	27	13	250
Availability of triage system (*n* = 251)	Yes	8 (18 %)	8 (17 %)	47 (40 %)	23 (85 %)	3 (23 %)	89 (35 %)
	No	35 (78 %)	37 (77 %)	67 (57 %)	4 (15 %)	7 (54 %)	150 (60 %)
	Don’t Know	2 (4 %)	3 (6 %)	4 (3 %)	0 (0 %)	3 (23 %)	12 (5 %)
	Total	45	48	118	27	13	251
Availability of hand hygiene (*n* = 252)	Yes	24 (53 %)	27 (56 %)	100 (84 %)	27 (100 %)	7 (54 %)	185 (73 %)
	No	17 (38 %)	13 (27 %)	16 (13 %)	0 (0 %)	4 (31 %)	50 (20 %)
	Don’t Know	4 (9 %)	8 (17 %)	3 (3 %)	0 (0 %)	2 (15 %)	17 (7 %)
	Total	45	48	119	27	13	252
Hand hygiene performed (*n* = 245)	Yes	18 (42 %)	24 (51 %)	93 (80 %)	25 (96 %)	5 (38 %)	165 (67 %)
	No	18 (42 %)	10 (21 %)	13 (11 %)	1 (4 %)	6 (46 %)	48 (20 %)
	Don’t Know	7 (16 %)	13 (28 %)	10 (9 %)	0 (0 %)	2 (15 %)	32 (13 %)
	Total	43	47	116	26	13	245

The level of awareness among infected HCWs about IPC and the availability of IPC facilities and policies in the health facilities where they worked at the time of their infection provide insight into the factors contributing to the occurrence of EVD infection among HCWs. A significant percentage of infected HCWs reported having been trained in IPC prior to their infection (Table 5). Of those who were trained, 69 % had received only basic IPC training and 31 % were trained as part of their general medical or nursing education. Furthermore, 60 % of the trained HCWs said they had been trained during the outbreak. Many respondents reported an IPC policy in place at their workplace at the time of their infection, and a large percentage reported available hygiene stations or facilities. A few respondents reported a functional triage system at their facility. However, several of the infected HCWs working in a hospital setting said that there were no IPC policies at their workplace (Table 5).

About half the DMOs and Matrons interviewed stated that they were unaware of a national IPC policy at the onset of the outbreak but that standard precautions were being taken in many of their health care facilities. Almost all interviewees identified district hospitals and EVD holding centres as the most difficult places to implement IPC measures aimed at ensuring HCW safety. The interviewees perceived common factors contributing to HCW infection in their districts to be the following: “negligence” (defined as non-adherence to basic IPC rules) and “overconfidence” (defined as a feeling of knowing the rules despite the opposite being true) of HCWs, both often resulting in breaches in IPC protocol; inadequate supervision; delayed and inadequate IPC training; inadequate supplies of IPC materials; poor triage systems at their health facilities. All interviewees stated that regular refresher IPC training, the availability of standard IPC protocols at all health facilities, strong support supervision, adequate provision of standard holding and treatment centres, uninterrupted supplies of IPC materials, and regular IPC audits would help prevent HCW infections in the current and future EVD outbreaks.

## Discussion

EVD infection among HCWs is a major concern in relation to both the welfare of HCWs and sustainability of health care services during outbreaks. It is therefore important to understand the scale of EVD acquisition by HCWs and contributing factors, to assist in the development of appropriate prevention strategies. This study represents a contribution toward that goal. A key finding of the study is that most HCW infections are associated with general health care and home settings but not with dedicated EVD settings. This has clarified the hitherto widely held belief that HCWs working in dedicated EVD facilities are at especially high risk compared with other HCWs. This finding should provide substantial reassurance to HCWs that measures in place at dedicated EVD facilities generally provide substantial protection when fully adhered to. This should in turn encourage more HCWs to volunteer for work in dedicated EVD isolation units. This result may also help alleviate the significant stigmatisation of HCWs working in such EVD facilities in Sierra Leone, which includes family and community rejection, isolation, and violence [14, 15].

HCW infections continue to be reported during this outbreak, despite initiatives intended to manage the risks. These initiatives include establishment of dedicated EVD isolation units, basic IPC training of health workers, and provision of PPE. This situation could be attributed to a number of reasons. First, a sizable percentage (34 %) of infected HCWs interviewed had not been trained in basic IPC at the time of their infection. Second, among those who were trained, more than 31 % had only been trained as part of their general medical or nursing education, which meant that their knowledge and skills in IPC may have been rudimentary because training was received a long time ago. Third, basic IPC training, which was intensified in September 2014, may have had a limited impact on the behaviour of trained HCWs. These trainings were typically one-off events in classroom settings, which are not comparable to conditions in the red zone of an actual EVD isolation unit or hospital setting. It is now generally recognised that such classroom education sessions have limited impact on HCW behaviour and organisational culture regarding IPC. In the absence of effective, ongoing audit programmes and supervisory structures in the workplace, non-adherence to basic IPC practice is commonplace, even in well-resourced settings. A sizable percentage of HCWs reported that the body part that had been contaminated was the hands, despite having performed hand hygiene at the time of exposure. The fact that these HCWs nevertheless became infected may indicate inappropriate hand hygiene technique.

The present findings show that hospitals and health centres were the most difficult health care settings for ensuring patient and HCW safety during the EVD outbreak. Factors that contributed to this vulnerability include weak management systems that resulted in frequent stock out of essential IPC material supplies, failure to identify the patients most likely to have transmissible infection (poor triage), and inadequate IPC facilities and training. There is also a lack of monitoring and supervision to support good IPC practice in many of the facilities. The observed high incidence of HCW infection in home settings may be attributed to a number of factors. In resource-poor African communities, self-medication and home management of minor ailments is common. In such settings, the first point of call for treatment is usually the home of relatives, friends, or close associates who are HCWs. Furthermore, private medical practice (often under suboptimal clinical conditions) to supplement personal income may have been a factor in HCW infection during the outbreak. Improved basic training in IPC as a core component of HCW training can be expected to enhance HCW capacity to recognise situations in which they are dealing with patients that may pose a high risk of infection, thereby enabling the HCWs to manage the risk more effectively, even in an informal setting.

Although a sizable proportion of the infected HCWs in the study were aware of the time and incident that resulted in their exposure, late presentation of infected HCWs to treatment facilities was a major issue. Delayed presentation could again be linked to the powerful stigma associated with EVD. Unpublished observations show that HCWs tend to hide their symptoms, perhaps for fear of ending up in the suspect or confirmed ward of an EVD treatment centre where conditions may not be comfortable for them, or fear of losing their jobs and source of income (mainly among junior cadres of HCWs).

HCW acquisition of EVD signifies basic deficiencies in implementation of and adherence to core IPC practices. Furthermore, the present study highlights the challenges associated with control of nosocomial transmission of pathogens in sub-Saharan African settings where health systems and basic IPC capacities are weak or lacking. The loss of nearly 10 % of HCWs in Sierra Leone is a major detriment that undermines the system and generates a vicious cycle of inadequate health care, increased nosocomial infection, and further weakening of the system. This produces a situation that will take years to rebuild [9]. Apart from the severe impact on HRH demonstrated by the study, the outbreak has also negatively affected health service delivery, health care financing, and governance. Bolkan et al. described a 50 % reduction in major surgeries and 70 % reduction in-patient admissions between May and October 2014 in Sierra Leone, likely owing to closure of health facilities because of the Ebola outbreak [16]. Delamou et al. [17] also attributed the reduction in reproductive health services in EVD-affected countries to desertion of health workers from health facilities, owing to fear of infection. It is important to note that the pre-Ebola health system situation in Sierra Leone was dire [18]. The current outbreak has further weakened the already fragile health care system in the country.

### Study limitations

Our study is subject to a number of limitations. The first is recall bias, which is an inherent limitation of a retrospective study. This is magnified by the traumatic experiences of survivors that may have affected their responses. The second limitation was the use of indirect sources, such as next of kin and professional colleagues of HCWs, in cases where HCWs enrolled in the study had died by the time of initial data collection. The third limitation is related to incompleteness of the national VHF database, so that we may not have identified and interviewed all infected HCWs during the study period. These limitations raise potential data quality problems. However, these were addressed through pre-testing of the questionnaire, in-depth training of the data collectors, peer review of completed questionnaires, and follow-up interviews when necessary. Fourth, we were unable to calculate reliable rates (such as attack rates), because of the unavailability of accurate denominators (exact numbers of HCWs at the time of the study). Finally, reasons for delay in seeking health care were not included in the study questionnaire; insight into the reasons for this was therefore collected during the key informant interviews and from our field observations.

## Conclusions

In conclusion, our study demonstrates that the risk of acquiring EVD applies to HCWs working in both EVD isolation units and general health care settings. We confirm the findings of other studies that nursing staff are a high-risk group for EVD infection [5]. The study also points to engagement of HCWs in unregulated private health care delivery outside formal health care settings as a risk. It is clear that workers in dedicated EVD facilities represent a small minority of those affected. Therefore, an effective response to reduce HCW infection is strong emphasis on reinforcing IPC capacity in general and in informal health care settings [19, 20]. In addition to reducing the impact of diseases such as EVD on HCWs, building IPC capacity will generally be of great benefit to the safety of patients and HCWs. One aspect of this is sensitising HCWs about the similarities between early clinical features of EVD and other endemic infectious diseases (such as malaria) that do not represent a similar risk. Such training should be comprehensive and focus on providing HCWs with hands-on experience in actual health facility settings. Ineffective health systems, which are prevalent in sub-Saharan African countries where EVD outbreaks occur, present a major challenge to achieving zero risk of nosocomial transmission of EVD. However, thorough understanding of the risk factors associated with transmission of the disease among HCWs early in the course of an outbreak and prompt instatement of remedial actions are likely to substantially protect HCWs against EVD and other virulent pathogens. The role of strong health systems in reducing nosocomial infection of HCW cannot be overemphasised, and should be integrated into all future EVD preparedness activities.

Based on our findings, we give two main recommendations aimed at preventing and reducing HCW infections during the current and future EVD outbreaks. First, clinical audit systems for real-time supervision, monitoring and evaluation of HCW infections should be established now and early in the course of future EVD outbreaks. Such systems will assure early detection, investigation, documentation, and response to nosocomial infection of HCWs. Second, a minimum standard of IPC practice that includes implementation of standard and transmission-based precautions should be defined and implemented. This process should involve development of a cadre of IPC specialists who can support implementation of appropriate IPC/patient safety policies, strategies, and guidelines. This in turn must be supported by uninterrupted supplies of basic IPC materials, such as hand hygiene products, gloves, aprons, and disinfectants to all health facilities in Sierra Leone.

## Abbreviations

CCC: Community Care Centre
CDC: Centers for Disease Control and Prevention
CFR: Case fatality ratio
DHMT: District health management team
DMO: District medical officer
EBOV: Ebola virus
EVD: Ebola virus disease
HCW: Health care worker
HRH: Human Resources for Health
IPC: Infection prevention and control
MOHS: Ministry of Health and Sanitation
PPE: Personal protective equipment
SOP: Standard operating procedure
VHF: Viral haemorrhagic fever
